# All over and Doing Well

**Published:** 1915-02

**Authors:** Mary Corner Raymond


### ALL OVER AND DOING WELL.

BY MARY CORNER RAYMOND.

Unaccustomed to politics and the "political game," the dentists of Oregon have come through an election campaign in triumph—not because they "played politics," but because they worked with fervor in what they believed to be a welfare movement, and not merely a political campaign.

The Dentistry Bill was defeated by a State majority of about 18,000. Next to the Prohibition Constitutional Amendment, the Dentistry Bill received more votes than any other of the 29 measures on the ballot. With an unusually long ballot, and such matters before the people as the death penalty, taxation measures and work-hour bills, the large vote on "341 X No" shows the widespread interest in the issue, and, furthermore, points the way to further endeavor on the part of the dental profession of this State.

A canvass of the ballots by counties shows that the vote was heaviest against the measure in those counties where the enthusiasm of the dentists ran highest and the hardest local work was done—another indication that the fight was won by work rather than luck. Those who were in the midst of the fray know it was "strenuous." "I believe we did the hardest work on the least money of any of the campaigns," said Dr. Millard C. Holbrook, Chairman of the Publicity Committee, after the election was over and the expenses of other campaign organizations and the election results were compared.

Owing to the false cry of "Trust" by the opposition and the long-settled prejudice of the press because dentists as a profession do not do general advertising, there was more to overcome than was at first anticipated. Nevertheless, in spite of the voluminous advertising done by the supporters of the bill, and the attempt to buy editorial support by big advertising contracts, more than fifty papers of the State took an independent stand, regardless of gain, and came out boldly for clean, honest dentistry and the upholding of the standard of the profession.

The general plan of organization and campaign are familiar enough to those in active touch with the work of the past four months to make unnecessary here a review of the methods followed. While there may have been some mistakes made and at times there were slight differences of opinion, it is believed by those who gave the most time to the campaign that the plan of a central organization, with the work throughout the State conforming in general with the suggestions of campaign headquarters, was the most effective system.

And, also, while there may not have been perfect unanimity on questions of policy, the consensus of opinion favored an impersonal fight, with the measure and the standard of dentistry as the only issue. The last few days of the campaign the personal issue was forced by the supporters of the bill. Their malicious and intentionally false charges and misleading lies compelled a refutation. The complete ignoring of the "enemy" was generally commended by the laity and the scheme for free publicity was frustrated.

Not even "the ransomed" will ever know the self-sacrifice of a number of dentists who almost sweat blood to save the name of dentistry in Oregon. No contribution in dollars and cents could measure what they gave in time and strength. They practically "shut up shop" and worked day and night in the campaign. It was literally "harder than pulling teeth."

The day is saved—today, perhaps, not tomorrow. It is agreed that it is "up to" the profession to hold the ground by continuing and extending the educational work that has been carried on for the last four or five years.

Eugene dentists are giving lectures in the schools. Pendleton dentists are planning a free dental clinic. A

recent editorial in a Baker paper points out the need of a free clinic for the school children of that city and emphasizes the importance of mouth hygiene. Portland has such welfare work under way as the municipal free dental clinic, the examination of the mouths of children at the Parents' Educational Bureau, under the auspices of the Oregon Congress of Mothers, and a toothbrush drill in one of the public schools.

The profession has been misunderstood and misrepresented. Every dentist knows that the charge of a "dental trust" is malicious and false. A part of the present need is to prove this to the public mind.

It has been said, "The knowledge of the dentist and the physician belongs to the world." While the gospel of service is freely preached among dentists, the public has not yet come to understand that the profession is humanitarian in its purposes.

With interest now keen, the golden opportunity is here for the dentists of Oregon to enlarge their educational work, and by a greater welfare movement continue to hold the faith and confidence of the people.—*Northwestern Dental Journal.*

